# Ginkgolide attenuates memory impairment and neuroinflammation by suppressing the NLRP3/caspase-1 pathway in Alzheimer’s disease

**DOI:** 10.18632/aging.205072

**Published:** 2023-10-03

**Authors:** Guang-Zhi Liu, Tian-Tong Niu, Qian Yu, Bao-Lei Xu, Xiao-Qing Li, Bo-Yi Yuan, Guo-Bin Yuan, Ting-Ting Yang, Hui-Qin Li, Yi Sun

**Affiliations:** 1 Department of Neurology, Beijing Anzhen Hospital, Capital Medical University, Beijing 100029, China; 2 Beijing D.A. Medical Laboratory, Beijing 102600, China; 3 National Clinical Research Center for Geriatric Diseases, Xuanwu Hospital, Capital Medical University, Beijing 100053, China; 4 Research and Development Centre, Chengdu Baiyu Pharmaceutical Co., Ltd., Chengdu 611130, China

**Keywords:** Alzheimer’s disease, amyloid beta, ginkgolide, microglia, NLRP3 inflammasome

## Abstract

The NLRP3 inflammasome is involved in the neuroinflammatory pathway of Alzheimer’s disease (AD). The aim of this study is to explore the roles and underlying mechanisms of ginkgolide (Baiyu®) on amyloid precursor protein (APP)/presenilin 1 (PS1) transgenic mice and a murine microglial cell line, BV-2. In the present study, the APP/PS1 mice were administered with ginkgolide, followed by a Morris water maze test. The mice were then euthanized to obtain brain tissue for histological and Aβ analysis. Additionally, BV-2 cells were pretreated with ginkgolide and then incubated with Aβ1–42 peptide. NLRP3, ASC, and caspase-1 mRNA and protein expression in brain tissue of mice and BV-2 cells were quantified by real-time PCR and western blotting, as well as reactive oxygen species (ROS) production, interleukin (IL)-1β and IL-18 levels by lucigenin technique and ELISA. Compared with the APP/PS1 mice, ginkgolide-treated mice demonstrated the shortened escape latency, reduced plaques, less inflammatory cell infiltration and neuron loss in the hippocampi of APP/PS1 mice. The levels of NLRP3, ASC, caspase-1, ROS, IL-1β, and IL-18 were also decreased in the brain tissue of APP/PS1 mice or Aβ1–42-treated BV-2 cells following ginkgolide treatment. Ginkgolide exerted protective effects on AD, at least partly by inactivating the NLRP3/caspase-1 pathway.

## INTRODUCTION

Alzheimer’s disease (AD) is an irreversible degenerative disorder of the central nervous system. AD is characterized by aberrant amyloid beta (Aβ) accumulation in senile plaques and neurofibrillary tangles consisting of highly phosphorylated tau proteins [1–3]. Although the amyloid hypothesis, cholinergic hypothesis, and tau protein theory are widely accepted, mounting evidence indicates a key role of neuroinflammation. Specifically, the activated microglia and astrocytes reportedly secrete toxic substances and pro-inflammatory cytokines, causing the neuronal dysfunction and apoptosis that result in AD pathology [4–6].

The nucleotide-binding oligomerization domain-like receptor family pyrin domain-containing 3 (NLRP3) inflammasome is a pivotal player in the AD inflammatory pathways [7, 8]. As an intracellular, multimolecular complex found in microglia, the NLRP3 inflammasome contains an NLRP3 scaffold, adaptor protein apoptosis-associated speck-like protein containing a CARD (ASC), and procaspase-1. Under pathological conditions, abnormal Aβ aggregation trigger an inflammatory response followed by a subsequent formation and activation of NLRP3 inflammasome, which thus generates activated caspase-1 [2, 3] and initiates the maturation and secretion of interleukin (IL)-1β and IL-18. These processes further contribute to AD progression. Notably, recent research has shown promising results, demonstrating that specific NLRP3 inhibitors attenuated the NLRP3 inflammasome activity *in vivo,* reduced tau and Aβ levels, and diminished cognitive impairment [9–11]. Hence, therapeutic strategies that inhibit NLRP3 inflammasome signaling as a consequence of neuroinflammation may halt or even reverse AD progression [12, 13].

In the past decade, plant-derived bioactive natural products have become popular for the development of therapeutic drugs for AD owing to their neuroprotective, anti-inflammatory, antioxidant, anti-amyloidogenic, and anticholinesterase activities [14, 15]. A variety of Chinese herbal medicine extracts (e.g., baicalin, schisandrin, nootkatone, and resveratrol) may prove beneficial in the treatment of AD via inhibition of the NLRP3 pathway [16–18]. Furthermore, Ginkgo biloba extracts have attracted attention due to their efficacy in treating dementia and hence have been recommended for AD treatment [19]. The active compounds in GB are terpene trilactones, which consist of bilobalide and ginkgolides A–C, J–N, P, and Q. Increasing evidence suggests that ginkgolides and bilobalide have extensive neuroprotective properties that may effectively treat AD [20, 21]. Of note, several *in vitro* and *in vivo* studies have recently reported that ginkgolide B remarkably improved cognitive function in senescence-accelerated mouse (SAMP8) or enhanced microglial M2 polarization by suppressing NLRP3 inflammasome activation [22, 23].

With ongoing advancements in technology, a new drug of ginkgolide (Baiyu^®^, Baiyu Pharmaceutical Co., Ltd., Chengdu, China) comprising ginkgolide A–C, J, and bilobalide has recently been developed and approved for treating ischemic cerebrovascular disorder, but its treatment efficacy for AD remains uncertain. We previously performed an *in vitro* study to investigate the effects of ginkgolide (Baiyu^®^) using an AD cellular model (amyloid precursor protein (APP)/presenilin 1 (PS1) double-transfected human embryonic kidney 293 cell line) [24]. The product significantly enhanced cell viability, demonstrating its neuroprotective effects on AD by suppressing the nuclear factor kappa B (NF-*κ*B) signaling pathway through anti-apoptosis and anti-inflammation mechanisms. However, little is known about ginkgolide’s neuroprotective activities against AD and NLRP3 activation. In the present study, we observed the effects of ginkgolide on Aβ accumulation, NLRP3 inflammasome activity, neuronal loss, and learning and memory impairment in APP/PS1 transgenic mice and a murine microglial cell line, BV-2. Furthermore, we explored the mechanisms underlying its anti-neuroinflammatory activities.

## RESULTS

### Ginkgolide attenuated cytotoxicity in Aβ_1–42_-treated BV-2 cells

**Figure 1:**
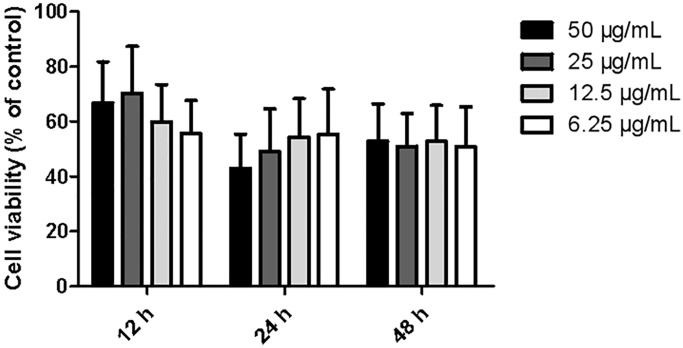
**Effects of different dosages of ginkgolide on BV-2 cell proliferative activity at different time points.** Data are presented as mean ± standard deviation (*n* = 3).

The proliferative activities in different dosage group (6.25, 12.5, 25, and 50 μg/ml) displayed an upward-downward-upward trend after 12, 24, and 48 hours of treatment with ginkgolide, respectively. Cell viability at 12 hours post-treatment was remarkably higher than that at 24 hours or 48 hours post-treatment. Furthermore, at 12 hours post-treatment, the cell viability at 25 μg/ml was remarkably increased compared with that of the other dosage groups (*P* < 0.01 and *P* < 0.01) (Figure 1). Based on these findings, 25 μg/ml and 12 hours post-treatment were selected as the optimal concentration and time point for cell proliferation, respectively.

### Ginkgolide inactivated NLRP3 inflammasome signaling in Aβ_1–42_-treated BV-2 cells

**Figure 2:**
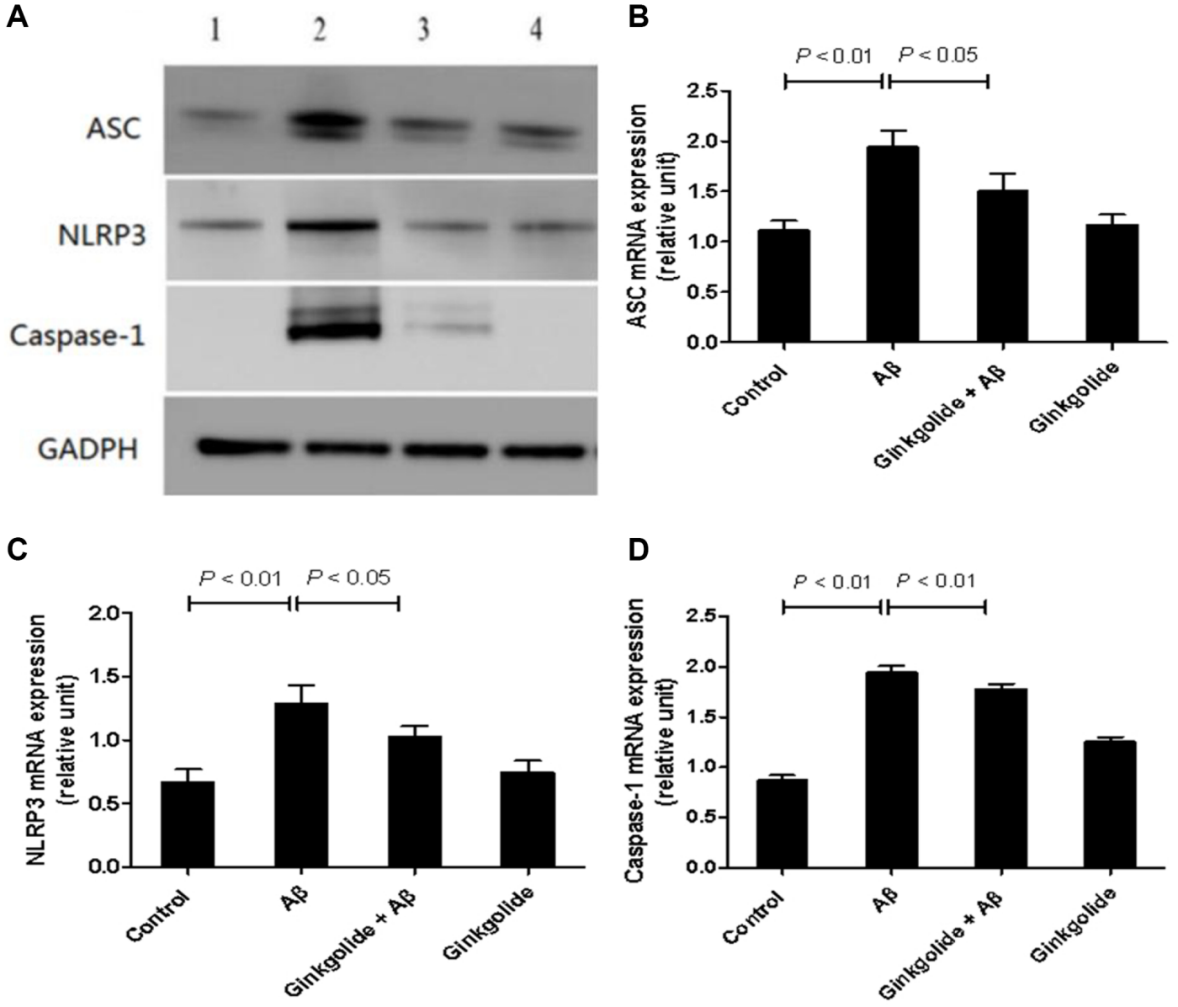
**Detection of intracellular protein expression of ASC, NLRP3, and caspase-1 in BV-2 cells by western blotting.** (**A**) Western blotting. Lane 1, control group (untreated); lane 2, Aβ group (treated with 2 μM Aβ1–42 for 12 h); lane 3, ginkgolide + Aβ group (pretreated with 25 μg/ml ginkgolide for 2 h followed by 2 μM Aβ1-42 for 10 h); lane 4, ginkgolide group (pretreated with 25 μg/ml ginkgolide for 12 h). (**B**–**D**) Effects of ginkgolide on intracellular protein expression of ASC, NLRP3, and caspase-1 in BV-2 cells. Data are presented as mean ± standard deviation. Abbreviations: Aβ: amyloid beta; ASC: apoptosis-associated speck-like protein containing a CARD; NLRP3: nucleotide-binding oligomerization domain-like receptor family pyrin domain-containing 3.

**Figure 3:**
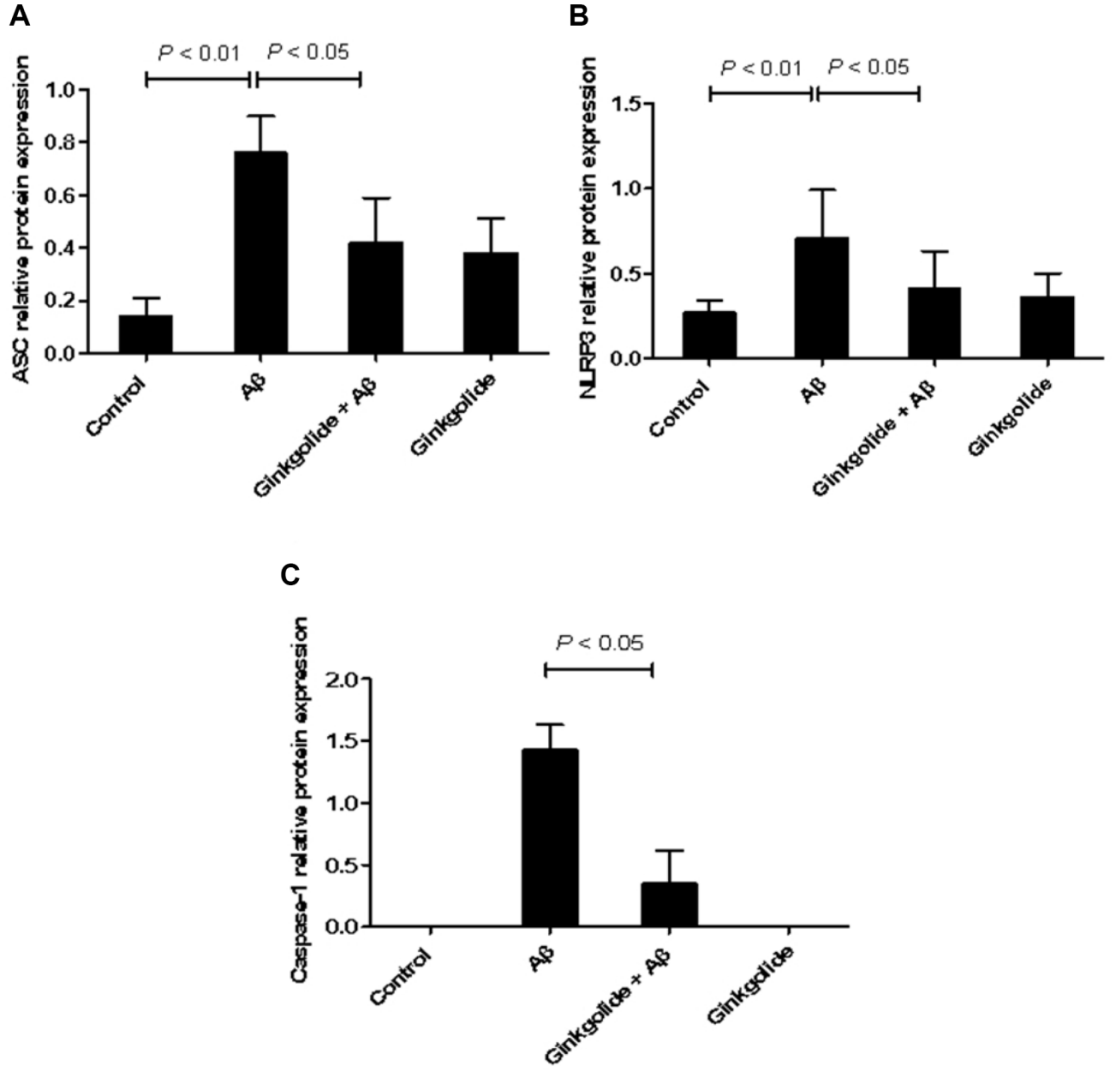
Effects of ginkgolide on mRNA expression levels of (**A**) ASC, (**B**) NLRP3, and (**C**) caspase-1 in BV-2 cells. Data are presented as mean ± standard deviation. Abbreviations: ASC: apoptosis-associated speck-like protein containing a CARD; NLRP3: nucleotide-binding oligomerization domain-like receptor family pyrin domain-containing 3.

To determine whether ginkgolide attenuated Aβ_1–42_-induced inflammatory responses by inactivating the NLRP3 inflammasome signaling pathway, BV-2 cells were pretreated with ginkgolide for 2 hours, and then stimulated with Aβ_1–42_ (2 μm) for 10 hours. As shown in Figures 2 and 3, the mRNA and protein expression of ASC, NLRP3, and caspase-1 were significantly upregulated in BV-2 cells after treatment with Aβ_1–42_. Pretreatment with ginkgolide, as we had expected, substantially decreased the mRNA and protein expression of ASC, NLRP3, and caspase-1 in BV-2 cells compared to Aβ_1–42_-treated group (*P* < 0.05 and *P* < 0.05, *P* < 0.05 and *P* < 0.05, *P* < 0.01 and *P* < 0.05).

### Ginkgolide suppressed production of pro-inflammatory cytokines and ROS in Aβ_1–42_-stimulated BV-2 cells

**Figure 4:**
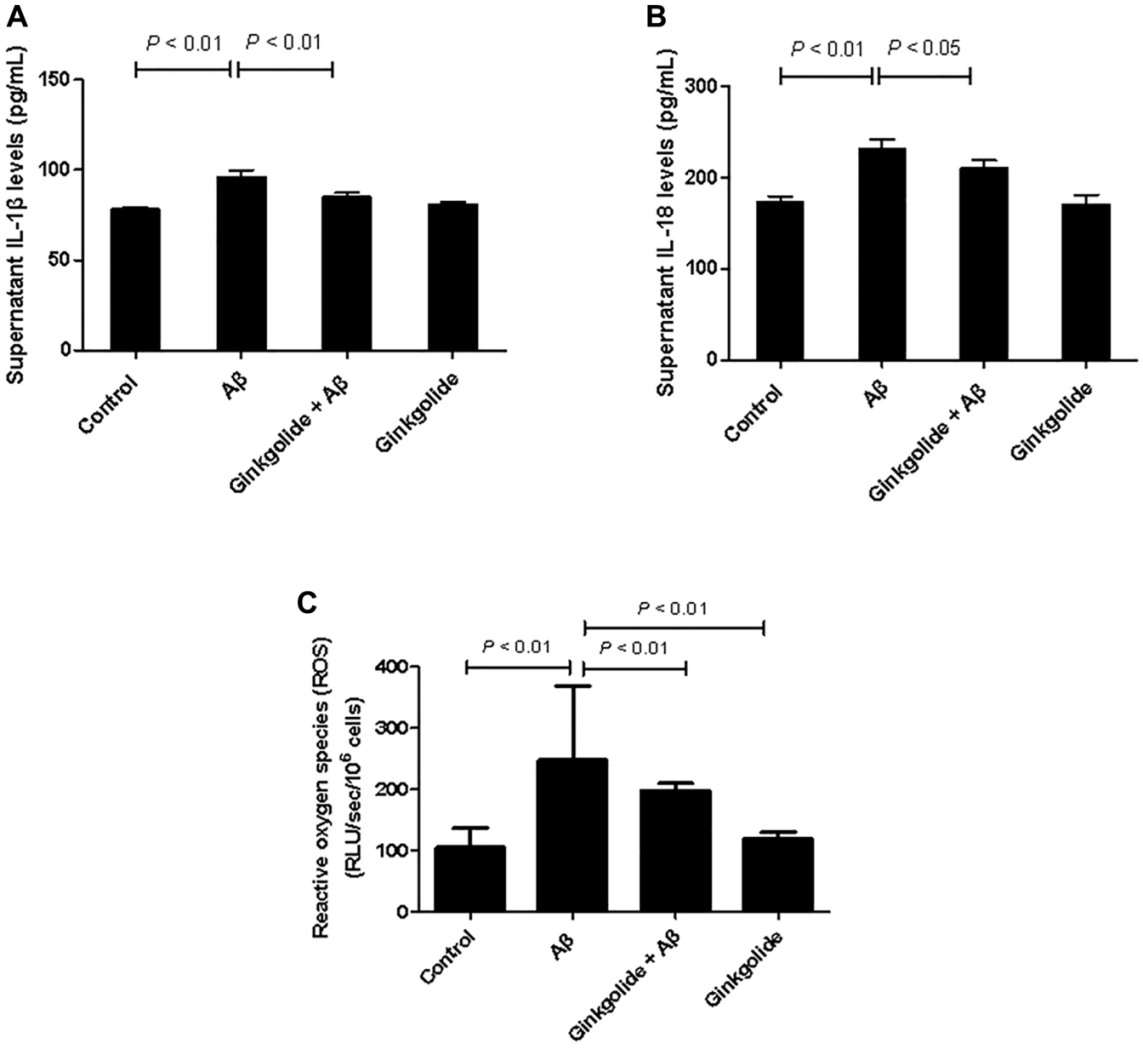
Effects of ginkgolide on supernatant levels of (**A**) interleukin (IL)-1β, (**B**) IL-18, and on (**C**) production of reactive oxygen species (ROS) in BV-2 cells. Data are presented as mean ± standard deviation.

To investigate the regulatory effects of ginkgolide on the production of pro-inflammatory cytokines in Aβ_1–42_-stimulated glial cells, BV-2 cells were pretreated with ginkgolide for 2 hours, followed by stimulation with Aβ_1–42_ for 10 hours. When compared to the control group, Aβ_1–42_-treated group showed remarkably increased supernatant expression of IL-1β and IL-18. However, these increases were significantly reduced by ginkgolide compared with Aβ_1–42_-treated group (Figure 4A, 4B, *P* < 0.01 and *P* < 0.05). ROS levels exhibited an increase in Aβ_1–42_-administrated cells compared with control cells (*P* < 0.01). Further, compared with those in Aβ_1–42_-treated cells, ROS levels were significantly decreased in either ginkgolide + Aβ group (*P* < 0.01) or ginkgolide-treated cells (Figure 4C, *P* < 0.01).

### Ginkgolide ameliorated impaired cognition and pathological alterations in APP/PS1 mice

**Figure 5:**
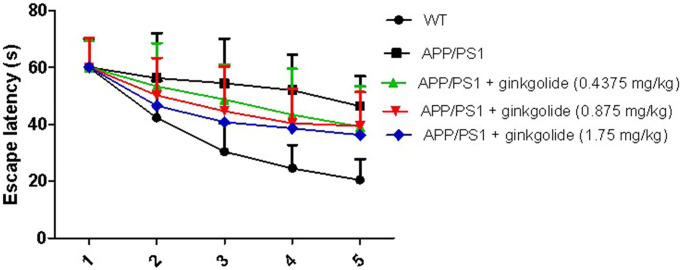
**Effects of different doses of ginkgolide on mice escape latency at different time points.** Data are presented as mean ± standard deviation (*n* = 5).

In the memory training experiment, the mean escape latencies in all groups of mice were remarkably reduced with increasing time. There was a marked increase in the escape latencies in the APP/PS1 group compared with the WT group (*P* < 0.01). Furthermore, at 5 days post-treatment with ginkgolide at doses of 0.4375, 0.875, and 1.75 mg/kg, the escape latency in each dosage group was significantly shorter than that of APP/PS1 group (*P* < 0.01), particularly at a dose of 1.75 mg/kg (*P* < 0.01) (Figure 5). Based on these results, post-treatment with ginkgolide (1.75 mg/kg) were selected as the optimal dosage for drug intervention study.

**Figure 6:**
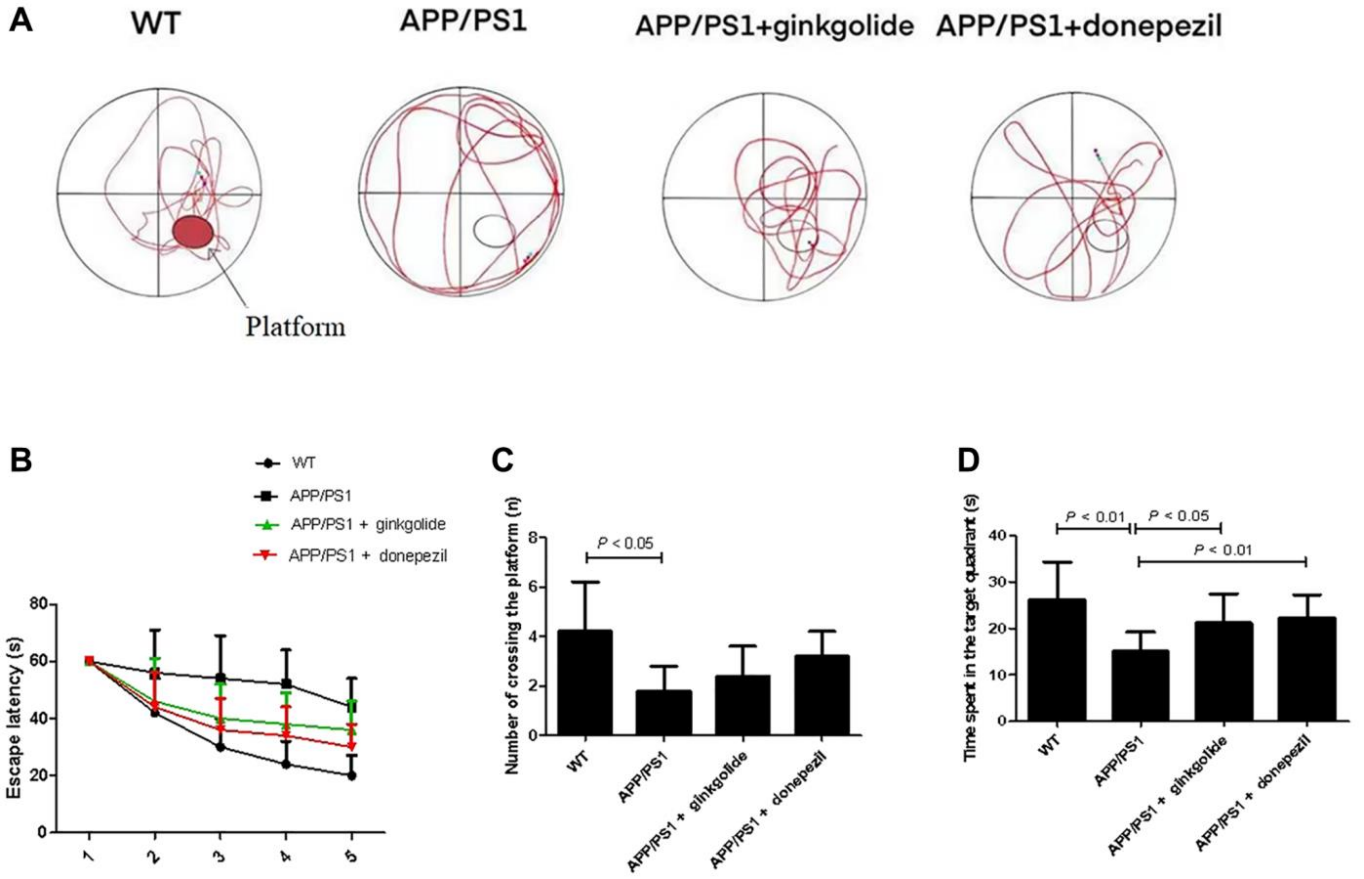
**Ginkgolide improved learning and memory impairment in APP/PS1 mice.** (**A**) Representative swimming track on day 5 of the Morris water maze test, (**B**) escape latency, (**C**) number of platform crossings, and (**D**) time spent in target quadrant on day 5 of the experiment. Abbreviations: APP/PS1: amyloid precursor protein/presenilin 1; WT: wild-type.

Behavioral performance was assessed using the Morris water maze method to determine the effect of ginkgolide intervention on memory deficits in AD mice (Figure 6A). Compared with the WT group, the APP/PS1 group exhibited prolonged escape latency; furthermore, escape latency was shortened in the ginkgolide group compared with that in the APP/PS1 group (Figure 6B, *P* < 0.05 and *P* < 0.05). Relative to the WT group, the number of platform crossings and time spent in the target quadrant were significantly decreased in the APP/PS1 group (*P* < 0.01 and *P* < 0.05), while the time spent in the target quadrant was remarkably elevated after ginkgolide or donepezil administration compared to APP/PS1 group (Figure 6C, 6D, *P* < 0.05 and *P* < 0.01).

**Figure 7:**
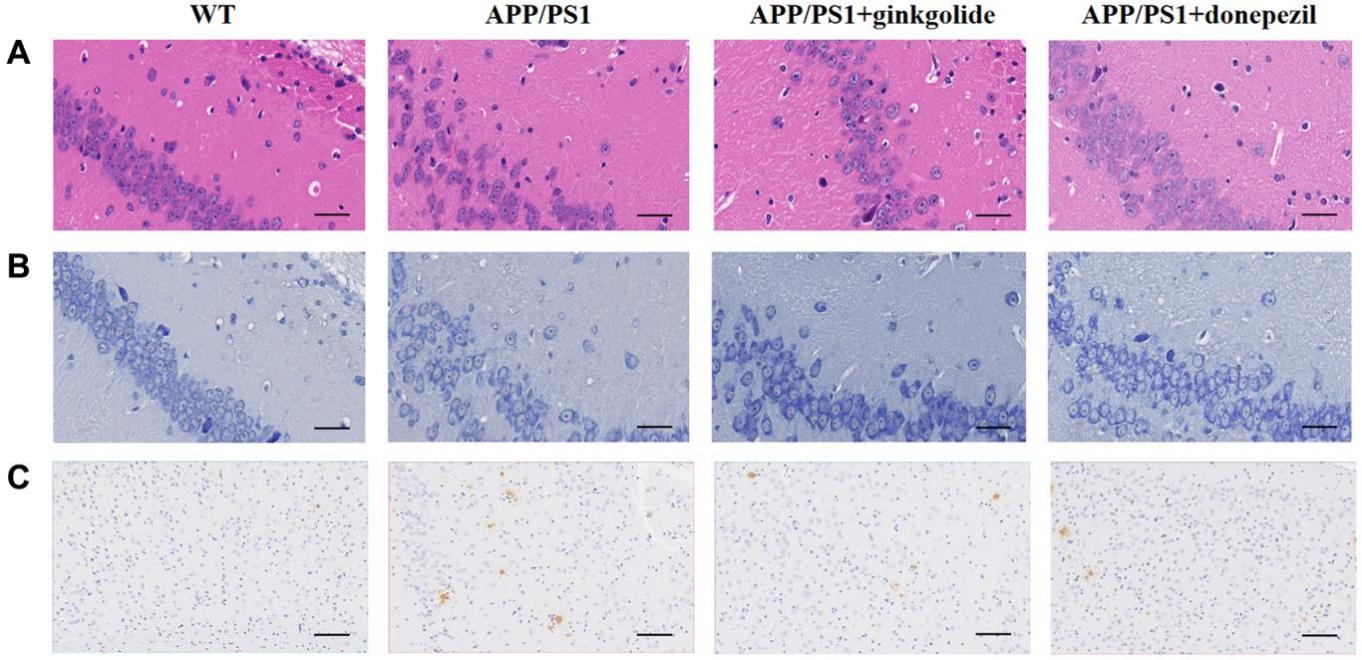
**Ginkgolides attenuated pathological alterations in the hippocampus and cerebral cortex of APP/PS1 mice.** Ginkgolides alleviated (**A**) inflammatory cell infiltration (H&E, ×400, scale bar = 50 μm), (**B**) neuron loss (Nissl staining, ×400, scale bar = 50 μm), and (**C**) the accumulation of Aβ in the brains of APP/PS1 mice shown by immunohistochemistry (×200; scale bar = 50 μm). Abbreviations: Aβ: amyloid beta; APP/PS1: amyloid precursor protein/presenilin 1; H&E: Hematoxylin and Eosin; WT: wild-type.

H&E and Nissl staining displayed greater inflammatory cell infiltration and neuron loss in the hippocampi and cerebral cortex of vehicle-treated APP/PS1 mice than in those of vehicle-treated WT mice and a reduction thereof with ginkgolide or donepezil administration (Figure 7A, 7B). Immunohistochemistry showed that vehicle-treated APP/PS1 mice had an over-accumulation of brain Aβ plaques, whereas these plaques were reduced in ginkgolide- and donepezil-treated AD mice (Figure 7C).

### Ginkgolide attenuated NLRP3 inflammasome activation in APP/PS1 mouse brains

**Figure 8:**
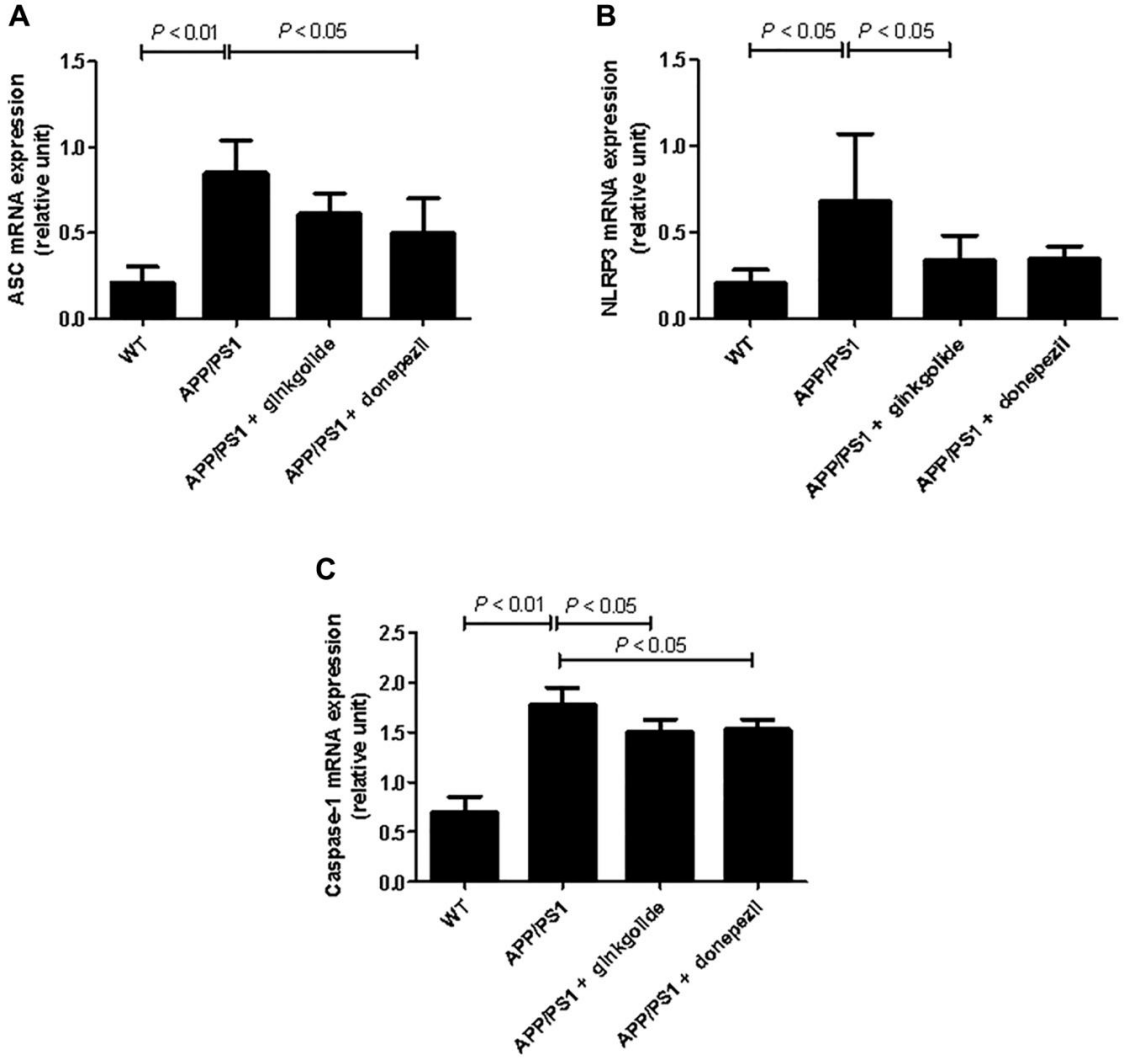
Effects of ginkgolide on the mRNA expression levels of (**A**) ASC, (**B**) NLRP3, and (**C**) caspase-1 in the brains (hippocampus and cerebral cortex) of APP/PS1 transgenic mice. Abbreviations: ASC: apoptosis-associated speck-like protein containing a CARD; APP/PS1: amyloid precursor protein/presenilin 1; NLRP3: nucleotide-binding oligomerization domain-like receptor family pyrin domain-containing 3; WT: wild-type.

**Figure 9:**
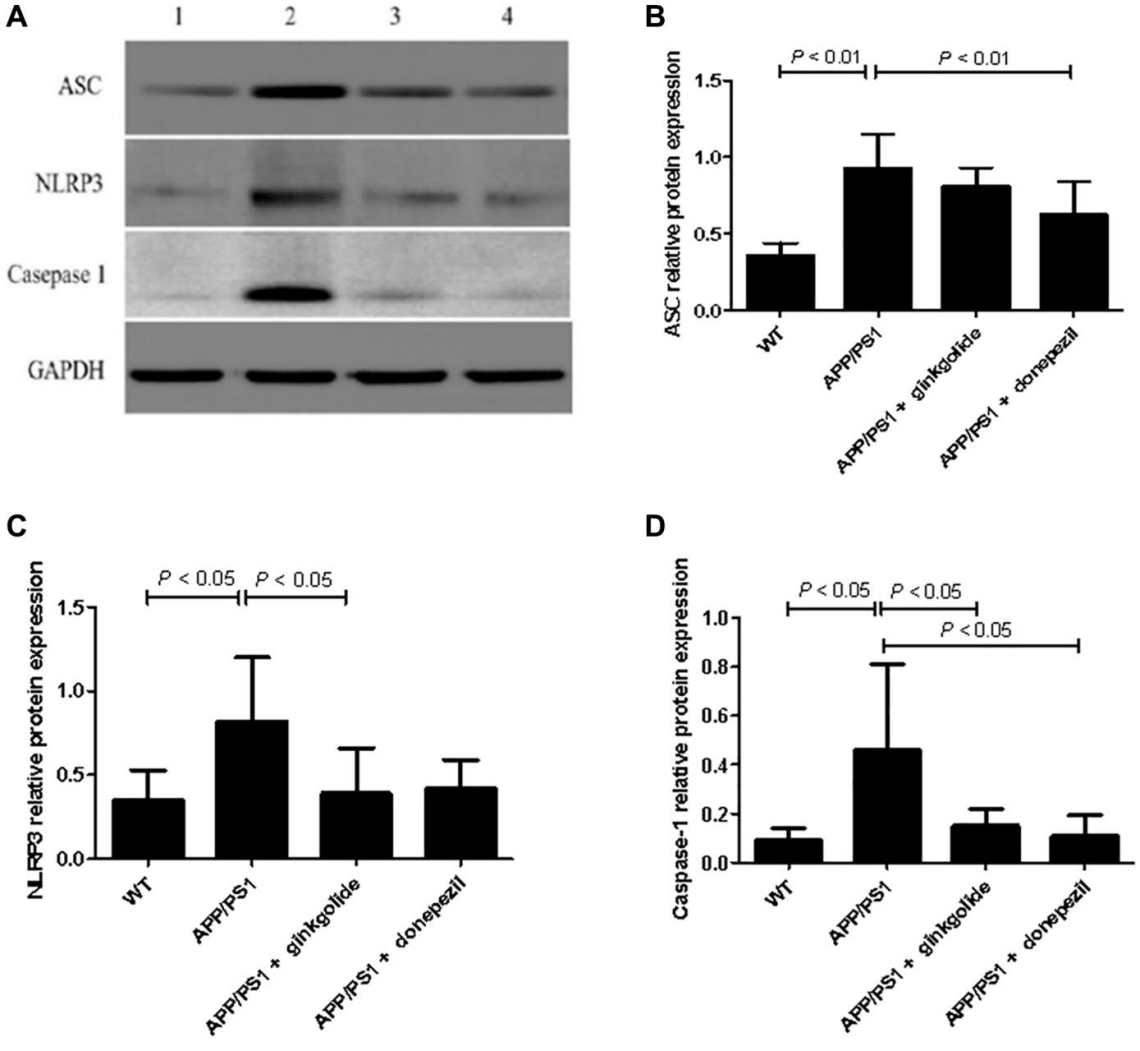
**Detection of intracellular protein expression of ASC, NLRP3, and caspase-1 by western blotting.** (**A**) Western blotting. Lane 1, WT group (treated with normal saline); lane 2, APP/PS1 group (treated with normal saline); lane 3, APP/PS1 + ginkgolide group (treated with 1.75 mg/kg ginkgolide); lane 4, APP/PS1 + donepezil group (treated with 0.65 mg/kg donepezil). (**B**–**D**) Effects of ginkgolide on the protein levels of ASC, NLRP3, and caspase-1 in the brains (hippocampus and cortex) of APP/PS1 transgenic mice. Data are presented as mean ± standard deviation. Abbreviations: ASC: apoptosis-associated speck-like protein containing a CARD; APP/PS1: amyloid precursor protein/presenilin 1; NLRP3: nucleotide-binding oligomerization domain-like receptor family pyrin domain-containing 3; WT: wild-type.

The mRNA and protein expression levels of NLRP3 inflammasome pathway molecules were detected in brain tissue using quantitative real-time PCR (Figure 8) and western blotting (Figure 9). As a result, relative to the WT group, the mRNA and protein levels of ASC, NLRP3, and caspase-1 increased in the hippocampal tissue of APP/PS1 group (mRNA: *P* < 0.01, *P* < 0.05, and *P* < 0.01; protein: *P* < 0.01, *P* < 0.05, and *P* < 0.05), but the mRNA and protein levels of NLRP3 were reduced in ginkgolide-treated group when compared to APP/PS1 group (*P* < 0.05 and *P* < 0.05). Moreover, expression levels of ASC and caspase-1 were lower in donepezil-treated group than those in APP/PS1 group (*P* < 0.05 and *P* < 0.05, *P* < 0.05 and *P* < 0.05).

### Ginkgolide decreased production of pro-inflammatory cytokines and ROS in APP/PS1 mice

**Figure 10:**
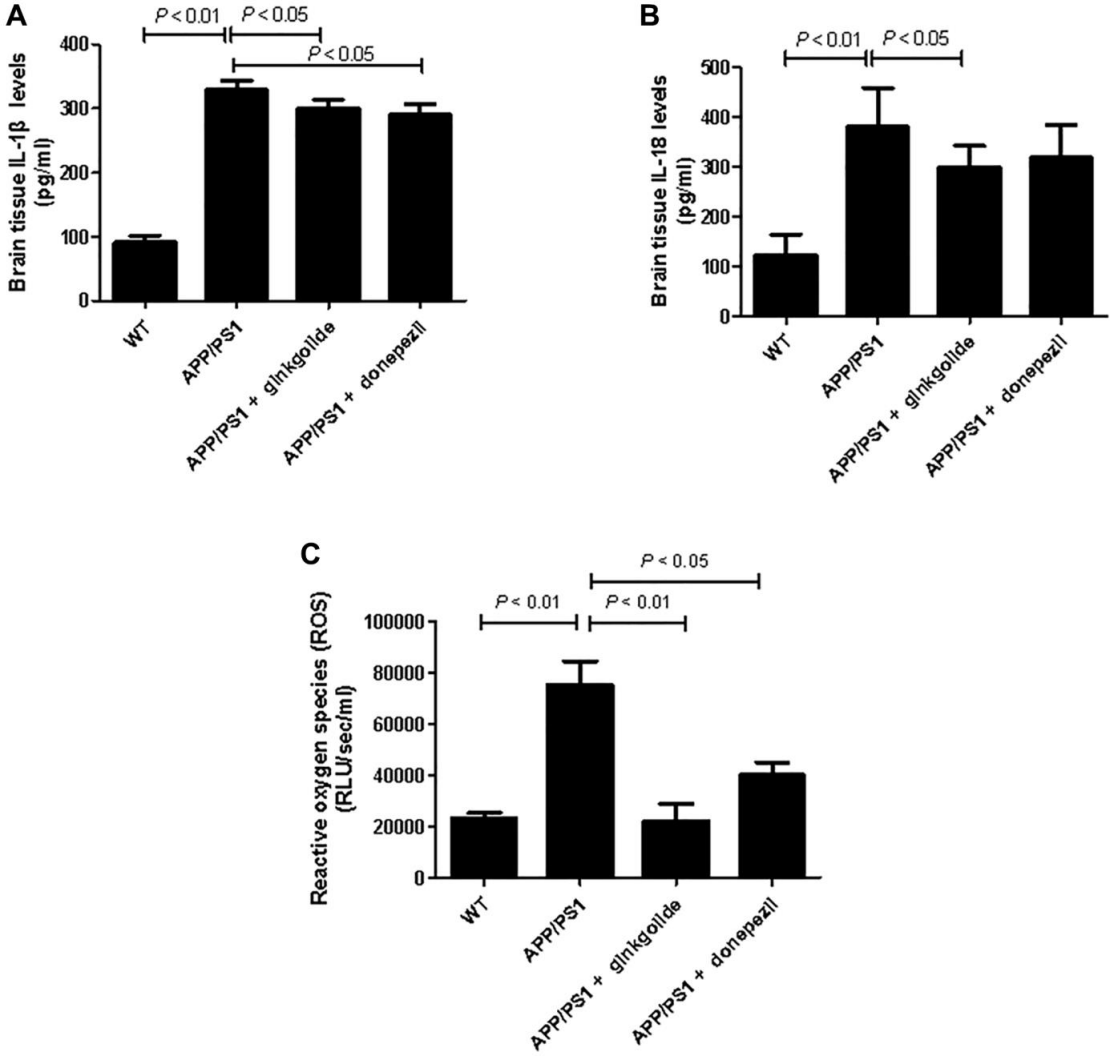
Effects of ginkgolide on (**A**) IL-1β, (**B**) IL-18, and (**C**) production of reactive oxygen species (ROS) in the brains (hippocampus and cortex) of APP/PS1 transgenic mice. Data are presented as mean ± standard deviation. Abbreviations: APP/PS1: amyloid precursor protein/presenilin 1; IL: interleukin; WT: wild-type.

To evaluate whether ginkgolide could reduce pro-inflammatory cytokines in the brains of APP/PS1 mice, we measured brain IL-1β and IL-18 protein levels. As shown in Figure 10, levels of IL-1β (Figure 10A) and IL-18 (Figure 10B) were significantly elevated in the cortex and hippocampus of the APP/PS1 group as compared to those in the WT group (*P* < 0.01 and *P* < 0.01). Relative to the APP/PS1 group, ginkgolide treatment significantly reduced IL-1β and IL-18 (*P* < 0.05 and *P* < 0.05), and donepezil decreased only IL-1β levels in the mice brain (*P* < 0.05). When compared to APP/PS1 group, ROS levels in the brain tissue of ginkgolide-treated or donepezil-treated mice were almost consistent with this group’s qRT-PCR and western blotting results (Figure 10C, *P* < 0.01 and *P* < 0.05).

## DISCUSSION

As a murine model of spontaneous AD, APP/PS1 mice develop cognitive and behavioral abnormality at 3–8 months of age [25, 26]. Increased deposition of Aβ and Aβ-associated neuroinflammation (e.g., microgliosis and astrogliosis) are observed in the cerebral cortex and hippocampi of mice [25, 27–29]. Various studies have confirmed that treatment with GB extract EGb 761 in murine AD models improves cognitive deficits and reduces Aβ aggregation or tau hyperphosphorylation in the brain [30–45], illustrating the therapeutic potential of ginkgolides and bilobalide in AD [46]. As expected, our *in vivo* experiments revealed that ginkgolide significantly ameliorated the learning and memory deficits and reduced Aβ deposition, inflammatory cell infiltration, and neuronal loss in the APP/PS1 mice brain. Our results, hence, substantiate the potential anti-AD effects of ginkgolide and its underlying neuroprotective and anti-inflammatory mechanisms.

Presently, microglia-based neuroinflammatory events driven by Aβ include the release of proinflammatory cytokines (i.e., IL-1, IL-6, and tumor necrosis factor-α (TNF-α)) and chemokines (i.e., monocyte chemotactic-1 and macrophage inflammatory protein-1) as well as ROS production. These events are major contributing factors in the pathogenesis of AD [47–50]. Notably, activation of the NLRP3 inflammasome in microglia and astrocytes within the central nervous system [12, 51] is believed to have a leading role in AD pathogenesis [7, 52–54]. Previous studies have shown that EGb exerts its anti-inflammatory effects through inhibiting microglial production of pro-inflammatory factors (e.g., prostaglandin E2, nitric oxide (NO), TNF-α, IL-1β, and IL-6) [55, 56]. In agreement with these findings, our *in vivo* experiments found that ginkgolide treatment resulted in decreased expression levels of NLRP3, ASC, and caspase-1, as well as reduced production of IL-1β and IL-18 in the AD mice brain, indicating the protective effects of ginkgolide through inactivation of the NLRP3/caspase-1 pathway, possibly in microglia. Indeed, similar results were observed in Aβ-treated BV-2 microglial cells following administration of ginkgolide, thereby supporting our hypothesis. Additionally, lower production of ROS was observed in Aβ_1–42_-stimulated, ginkgolide-treated BV-2 cells, suggesting that ginkgolide may promote a switch from M1 proinflammatory phenotype to M2 anti-inflammatory phenotype in BV-2 cells, as The M1 phenotype is critical for secretion of ROS, inducible nitric oxide synthase (iNOS), and pro-inflammatory factors (TNF-α, IL-1β and IL-6) to initiate proper inflammatory responses [57, 58]. On the other hand, changes in anti-inflammatory phenotype of the BV-2 cells should also be observed after the drug intervention, since several recent studies revealed that treatment with other drugs, such as fatty acid amide hydrolase inhibitor or hydroxysafflor yellow A, induced microglia polarization toward anti-inflammatory phenotype [59, 60]. Nonetheless, more efforts are required to investigate the state of the cells during drug administration in our future study.

Currently, the acetylcholinesterase inhibitors (AChEIs), such as donepezil and rivastigmine, have been approved to treat AD. Aside from their effects on AChE, AChEIs possess other biological activities, including suppression of Aβ plaque formation and brain inflammation [61–64]. Moreover, in this study we chose donepezil as anti-inflammatory control, rather than other typical medicines, such as Ibuprofen, mainly because the data regarding treatment of AD with Ibuprofen from epidemiological studies, clinical trials and meta-analyses in the past is still controversial [65]. Interestingly, several *in vitro* and *in vivo* studies have reported the anti-inflammatory effects of donepezil on various stimulus-induced inflammatory responses [66–71]. For instance, use of donepezil can inhibit Aβ-mediated upregulation of proinflammatory factors (e.g., nitric oxide (NO), iNOS, IL-1β, TNF-α) and suppress the p38/p65 signaling pathway in BV-2 cells, rat primary microglia, and mice brain [71]. Importantly, a recent study by Kim et al. illustrated the therapeutic potential of donepezil; in response to lipopolysaccharide- and Aβ-induced neuroinflammation, the drug suppressed AKT/mitogen-activated protein kinase, NLRP3 inflammasome, and NF-*κ*B/signal transducer and activator of transcription 3 signaling *in vitro* and *in vivo*. This suggests that donepezil could effectively treat neuroinflammation-associated diseases such as AD [63]. Consistent with these findings, our experiment found that after treatment with donepezil, down-regulated expression of ASC and caspase-1 occurred in the brains of AD mice, and decreased IL-1β and ROS was also observed, supporting a role for donepezil in modulating the NLRP3 inflammasome. However, unlike ginkgolide, no changes in NLRP3 expression were observed in donepezil-treated AD mice, despite downregulation of ASC and caspase-1 expression. Thus far, the effects of donepezil on Aβ-induced NLRP3 inflammasome pathways have not been well established. Hence, we speculate that donepezil might exert distinct modulatory action on the NLRP3 pathway, as AChE inhibition regulates the inflammatory response via the modulation of ACh levels and the activation of α7 nicotinic AChRs [71, 72]. Nonetheless, further research is needed to ascertain the exact roles of AChEIs.

The present study has several limitations. First, APP/PS1 mice were employed because this is a common animal model for AD; however, this model may not reflect all types of this disease [73]. Therefore, other AD animal models should be assessed to validate the effects of ginkgolide. Second, the sample size of animals included in this study was relatively small, and a larger sample size is required to confirm our results. Third, pro-inflammatory and anti-inflammatory response may coexist in neuroinflammatory process in AD [74, 75]; hence, further investigation is warranted to address this issue. Finally, while ginkgolide B has been investigated [22, 23], analysis of other components, such as ginkgolide A, C, J, and bilobalide, is needed to determine the role of these specific compounds in immunomodulation of the AD-associated NLRP3/caspase-1 pathway.

In conclusion, for the first time, we determined that ginkgolide exerted neuroprotection in a mouse model of AD, reduced Aβ deposition, and attenuated inflammatory cell infiltration and neuronal loss, thus improving cognitive function. Furthermore, ginkgolide prevented Aβ-induced neuroinflammation and ameliorated microglia-mediated neurotoxicity in BV-2 cells. The neuroprotective effect of ginkgolide may be mediated by its anti-neuroinflammatory activities, at least partially via the inactivation of the NLRP3/caspase-1 pathway. However, further studies on different animal models of AD and human clinical trials are warranted. Nonetheless, our findings pave the way for new treatment options for AD, and ginkgolide may emerge as a promising drug candidate for the treatment of this disease.

## MATERIALS AND METHODS

### Chemicals and reagents

Ginkgolide and donepezil were provided by Baiyu Pharmaceutical Co., Ltd. (Chengdu, China) and Eisai Co., Ltd. (Tokyo, Japan), respectively. Aβ_1–42_ was obtained from Shanghai Aladdin Biochemical Technology Co., Ltd. (Shanghai, China). BCA protein assay kit and 3-(4,5-dimethyl-2-thiazolyl)-2,5-diphenyl-2H-tetrazolium bromide (MTT) assay kits were purchased from Thermo Fisher Scientific (Waltham, MA, USA). Mouse IL-1β and IL-18 enzyme-linked immunosorbent assay (ELISA) kits were obtained from Multisciences (Lianke) Biotech (Hangzhou, China). Antibodies were obtained from several companies: anti-Aβ from Santa Cruz Biotechnology, Inc. (Santa Cruz, CA, USA); anti-NLRP3 from and anti-caspase-1 from Novus Biologicals (Littleton, CO, USA); anti-ACS from Thermo Fisher Scientific (Waltham, MA, USA); goat anti-rabbit and anti-mouse immunoglobulin G (H + L)-horseradish peroxidase (HRP) from Jackson ImmunoResearch Laboratories, Inc. (West Grove, PA, USA); and anti-glyceraldehyde-3-phosphate dehydrogenase (GAPDH) from Tianjin Ringpu Bio-Technology Co., Ltd. (Tianjin, China). TRIzol reagent was obtained from Tiangen Biotech Co., Ltd. (Beijing, China). RNAiso Plus, PrimeScript™ room temperature (RT) Reagent Kits with genomic (g) DNA Eraser, SYBR Premix Ex Taq (Tli RNase H Plus), and a DL 2,000 DNA Marker were purchased from Takara Biomedical Technology (Beijing, China). Electrochemiluminescence (ECL) kits were obtained from Pierce Biotechnology (Rockford, IL, USA).

### Cell culture and drug administration

Aβ_1–42_ was dissolved in double-distilled water at a concentration of 50 μM and incubated at 37°C for 5 days to promote fibrilization and aggregation. A BV-2 murine microglial cell line was obtained from Hanheng Biotechnology Co., Ltd. (Shanghai, China) and cultured in Dulbecco’s modified Eagle’s medium (Sigma-Aldrich, St. Louis, MO, USA) supplemented with 10% phosphate-buffered saline (PBS), 100 units/ml of penicillin, and 100 μg/ml of streptomycin. The BV-2 cells were cultured at 37°C in a humidified atmosphere containing 5% CO_2_. When the confluence of 70–80% was reached, cells were seeded into 6-well plates at a density of 5 × 10^4^ cells/ml and stimulated with ginkgolide at different concentrations (6.25, 12.5, 25, and 50 μg/ml, *n* = 3 each). Detection was carried out at 12, 24, and 48 hours post-treatment. In brief, MTT assay was conducted to determine the optimal time point and concentration for cell proliferation. Consequently, cells were treated with or without ginkgolide for 1 hour, followed by an incubation with or without Aβ_1–42_ for 12 hours. The experimental groups (*n* = 4 each) were: (1) control group (untreated); (2) Aβ group (treated with 2 μM Aβ_1–42_ for 12 hours); (3) ginkgolide + Aβ group (pretreated with 25 μg/ml ginkgolide for 2 hours followed by 2 μM Aβ_1–42_ for another 10 hours); and (4) ginkgolide group (pretreated with 25 μg/ml ginkgolide for 12 hours).

### Mice and drug administration

Eight-month-old male C57BL/6J wild-type (WT) and APP/PS1 transgenic mice sharing the same genetic background were obtained from the Model Animal Research Center of Nanjing University (Nanjing, China). APP/PS1 mice at 8 months of age were selected as subjects for drug intervention study, because animals already demonstrate Aβ pathology and memory deficits [76]. All mice were housed in a pathogen-free room with a 12-hour light/dark cycle and had ad libitum access to both feed and water. After 1-week adaptation, the mice were intraperitoneally administered 0.4375, 0.875, or 1.75 mg/kg ginkgolide twice a day for 60 days. Briefly, a Morris water maze test was performed to determine the optimal dosage for treatment. Based on these experiments, the APP/PS1 mice were randomly divided into three groups (*n* = 8 each): (1) normal saline (intraperitoneally administered 5 ml/kg normal saline twice a day for 60 days); (2) ginkgolide (intraperitoneally administered 1.75 mg/kg ginkgolide twice a day for 60 days); and (3) donepezil group (orally administered 0.65 mg/kg donepezil once a day for 60 days). Additionally, a control group comprising four healthy WT mice was intraperitoneally injected with 5 ml/kg normal saline for 60 days (*n* = 8). Upon completion of the behavioral tests, the mice were euthanized by cervical dislocation for brain tissue collection. All steps were performed to reduce pain, suffering and distress.

### Morris water maze test

Following drug administration, a Morris water maze test was performed (MT-200; Chengdu Taimeng Software Co., Ltd., Chengdu, China) and evaluated by the automated EthoVision^®^ XT 7.0 video-tracking system (Noldus Information Technology, Wageningen, The Netherlands). A 150 cm diameter circular pool was filled with 23 ± 1°C water and contained a 13 cm diameter platform 1 cm below the water surface. The animals of the four experimental groups (*n* = 4 each) were trained with space-learning tasks (≤60 seconds) four times a day for 5 consecutive days. At day 6, a probe trial was conducted for 60 seconds in the absence of a hidden platform. The escape latency (time to find the hidden platform in the Water Maze) [77, 78] and swimming path were recorded.

### Brain tissue collection

According to the completely randomized block design, mice in the four experimental groups (*n* = 4 each) were randomly chosen. All mice were deeply anesthetized with 2% sodium pentobarbital, transcardially perfused with normal saline and fixed with ice-cold 4% paraformaldehyde upon completion of behavioral analysis. The cerebral cortex and hippocampus were carefully removed, rapidly fixed in 4% paraformaldehyde at 4°C overnight and cryoprotected for 72 hours in 30% sucrose solution. Subsequently, the tissue was embedded in paraffin and cut into 5 μm standard sections for further morphological analysis. To perform biochemical assays (*n* = 4 each), the remaining mice underwent transcardial perfusion with normal saline, followed by dissection of the cortex and hippocampus. For western blotting experiments, brain samples were immediately stored at −80°C.

### Hematoxylin and eosin and Nissl staining

As previously described [79], the 5 μm-thick brain tissue sections were deparaffinized and rehydrated. Stepwise staining was done with hematoxylin and eosin (H&E) or Nissl dyes, and the sections were assessed under a light microscope (CKX41; Olympus, Tokyo, Japan).

### Immunohistochemistry

The brain tissue sections of four experimental groups were incubated with primary antibodies against Aβ at 1/1000 dilution at 4°C overnight (*n* = 4 each), and then washed with PBS. The sections were then incubated with HRP-conjugated anti-rabbit secondary antibody at 25°C for 1 hour, and further incubated with streptavidin-HRP complex (BestBio Science, Shanghai, China) at 25°C for another hour. Subsequently, the slices were counterstained by 5% 3,3′-diaminobenzidine tetrahydrochloride solution and hematoxylin (25°C for 5 minutes). Photomicrographs were acquired using an inverted fluorescent microscope (×40 and ×200 magnification; Olympus, Tokyo, Japan).

### Reactive oxygen species assay

Reactive oxygen species (ROS) production in the BV-2 cells or brain tissue of four experimental groups was detected by the lucigenin technique (*n* = 4 each). Briefly, the BV-2 cells were counted, and 1 × 10^7^ cells were homogenized in radioimmunoprecipitation assay (RIPA) buffer (PBS, 1% Nonidet P-40, 0.5% sodium deoxycholate, 0.1% sodium dodecyl sulfate (SDS), and a protease inhibitor cocktail) (Santa Cruz, Santa Cruz, CA, USA). For the brain tissue, samples were weighed and homogenized in 1:10 w/v RIPA buffer. After homogenization, both BV-2 cell and brain tissue samples were centrifuged at 12,000 revolutions per minute (rpm) for 20 minutes at 4°C. Once the supernatant was aspirated, the remaining cellular debris was discarded. The supernatant was incubated with lucigenin according to the manufacturer’s instructions (Genmed Scientifics Inc., Boston, MA, USA). The samples were allowed to equilibrate for 15 minutes, and then luminescence was measured every second for 10 seconds with a luminometer (Berthold Technologies, Oak Ridge, TN, USA). Luminescence was recorded as relative light units per second. An assay blank containing lucigenin but no homogenate was subtracted from the reading before data transformation. Measurement of each sample was repeated five times, and the average value was used for transformation of the data.

### Quantitative real-time polymerase chain reaction

RNAiso Plus was used to isolate and extract total mRNA from the mice brain samples or BV-2 cells of four experimental groups (*n* = 4 each) according to the manufacturer’s instruction. Reverse transcription of the mRNA (500 ng) was performed using a PrimeScript™ RT reagent kit with a gDNA Eraser. To quantify gene expression levels of NLRP3, ASC and caspase-1, 2 μL cDNA was amplified via real-time quantitative reverse transcription polymerase chain reaction (qRT-PCR) using the following primers: NLRP3 (forward, 5′-TGT CAG GAT CTC GCA TTG GT-3′; reverse, 5′-ATT GCT TCG TAG ATA GAG GTG TGT-3′); caspase-1 (forward, 5′-GTC TCA TGG TAT CCA GGA GGG-3′; reverse, 5′-TCA CCT TGG GCT TGT CTT TC-3′); ACS (forward, 5′-CCT GAG TAC AGC AGA GGT GGA-3′; reverse, 5′-CAC ACA AGG TAA CAA AGC AGT AGA-3′); and β-actin (forward, 5′-CCA TCT ACG AGG GCT ATG CT-3′; reverse, 5′-CTT TGA TGT CAC GCA CGA TT-3′) as an endogenous control. qRT-PCR was done using SYBR^®^ Premix Ex Taq™ II in a real time thermocycler (iQ5, Bio-Rad, Hercules, CA, USA). All the amplifications were conducted in triplicate for each sample. Amplification was performed under the following conditions: 95°C for 30 seconds, 40 cycles at 95°C for 5 seconds, and 60°C for 40 seconds. The relative mRNA levels were analyzed using the 2−ΔΔCt method as detailed by the manufacturer (Technical Bulletin 2; Applied Biosystems, Waltham, MA, USA).

### Western blotting

Briefly, after homogenization of the mouse brain tissue and BV-2 cells in ice-cold extraction reagent, centrifugation of the extract was performed at 4°C at 10,000 rpm for 10 minutes, and the supernatant of four experimental groups was collected (*n* = 4 each). Bicinchoninic acid protein assay was used to measure protein concentration. Equivalent amounts of protein (13 μg) for each sample were denatured by boiling at 95°C for 5 minutes, and separated using 12–15% SDS-polyacrylamide gels electrophoresis. Following electrophoresis, proteins were transferred to nitrocellulose membranes, which were blocked in 5% nonfat dry milk at room temperature for 30 minutes and incubated with primary antibodies against NLRP3, caspase-1, ACS, and GAPDH (all dilution ratios: 1:5000) at 4°C overnight. Thereafter, the membranes were washed with tris-buffered saline containing 0.1% Tween 20, and incubated with corresponding secondary antibodies (1:5000) at room temperature for 2 hours. Blots were visualized using an enhanced ECL detection kit, and quantified using ImageJ software (National Institutes of Health, Rockville, MD, USA).

### Enzyme-linked immunosorbent assay

To measure IL-1β and IL-18 levels in APP/PS1 mouse brains, the cortical and hippocampal tissues of mice were homogenized in cold homogenization buffer containing a protease inhibitor cocktail. After centrifugation at 12,000 rpm for 15 minutes at 4°C, the supernatant of four experimental groups was collected (*n* = 4 each). Additionally, after treatment, the BV-2 cell culture media of four experimental groups were collected (*n* = 4 each) and centrifuged at 8,000 rpm for 15 minutes at 4°C. The concentration of mouse IL-1β and IL-18 was detected using an ELISA kit according to the manufacturer’s protocols. Absorbance was measured at a wavelength of 450 nm using a microplate absorbance reader (Thermo Scientific Multiskan MK3, Shanghai, China).

### Statistical analyses

Statistical analyses were done using GraphPad Prism 8.0.1 (GraphPad Software, Inc., San Diego, CA, USA). All data are expressed as mean ± standard deviation or median and range. Normally distributed data were evaluated by one-way analysis of variance (ANOVA) using a Student–Newman–Keul’s post-hoc test. Non-normally distributed data were analyzed using Kruskal–Wallis test. A *p*-value < 0.05 was set as statistically significant.

### Data availability

The datasets generated during and/or analyzed during the current study are available from the corresponding author on reasonable request.
